# Treatment of calcium in hard water from acid sulfate soil and its utilization in a pre-designed cation exchange resin system for fluoride adsorption in water

**DOI:** 10.1039/d5ra01384d

**Published:** 2025-04-28

**Authors:** Trung Thanh Nguyen, Cam Tien Nguyen Thi, Bich Son Doan Thi, Nguyen Thi Thuy, Tri Thich Le, Phuoc Toan Phan, Le Ba Tran, Quynh Anh Nguyen Thi, Surapol Padungthon, Nguyen Nhat Huy

**Affiliations:** a Laboratory of Nanomaterial, An Giang University 18 Ung Van Khiem St, Dong Xuyen Ward Long Xuyen City An Giang Province Vietnam; b Vietnam National University Ho Chi Minh City Linh Trung Ward, Thu Duc City Ho Chi Minh City Vietnam; c Center for Energy and Environmental Materials, Institute of Fundamental and Applied Sciences, Duy Tan University 10C Tran Nhat Duat St, District 1 Ho Chi Minh City Vietnam; d Faculty of Environmental and Chemical Engineering, Duy Tan University 254 Nguyen Van Linh St, Thanh Khe District Da Nang City Vietnam; e Institute for Environment and Resources 142 To Hien Thanh St, Dist 10 Ho Chi Minh City Vietnam; f School of Chemical and Environmental Engineering, International University Quarter 6, Linh Trung Ward, Thu Duc City Ho Chi Minh City Vietnam; g Faculty of Engineering – Technology – Environment, An Giang University 18 Ung Van Khiem St, Dong Xuyen Dist Long Xuyen City An Giang Province Vietnam; h Faculty of Environment and Natural Resources, Ho Chi Minh City University of Technology (HCMUT) 268 Ly Thuong Kiet St, Ward 14, Dist 10 Ho Chi Minh City Vietnam nnhuy@hcmut.edu.vn +84 901 964 985; i Department of Environmental Engineering, Khon Kaen University 123 Moo 16 Mittraphap Rd, Nai-Muang, Muang District Khon Kaen 40002 Thailand

## Abstract

In this study, a pre-designed cation exchange resin (CER) column was first tested to remove calcium in naturally contaminated water from acid sulfate soil for water supply purpose, and it was further used as a novel and natural calcium hydroxide-supported CER composite (NCHO/CER) for the treatment of fluoride in water. Results of SEM, EDX, FTIR, and XRD analyses confirmed the formation of calcium hydroxide nanoparticles in the CER structure. The fluoride adsorption capacity of NCHO/CER was systematically studied by varying the influencing factors, such as the reaction time, pH level, adsorbent mass, initial concentration, and temperature. The adsorption kinetics followed a pseudo-second-order model, while the isotherm curves fitted to the Freundlich model, indicating the occurrence of multilayer adsorption on the adsorbent surface. Moreover, NCHO/CER showed the ability not only to be reused after multiple cycles but also to remove fluoride from actual tap water and groundwater, proving that NCHO/CER is effective for the recovery and reuse of contaminated calcium for fluoride treatment in water.

## Abstract 2

Calcium in hard water from acid sulfate soil as a resource to synthesize an efficient adsorbent for fluoride treatment in water.

## Graphical abstract

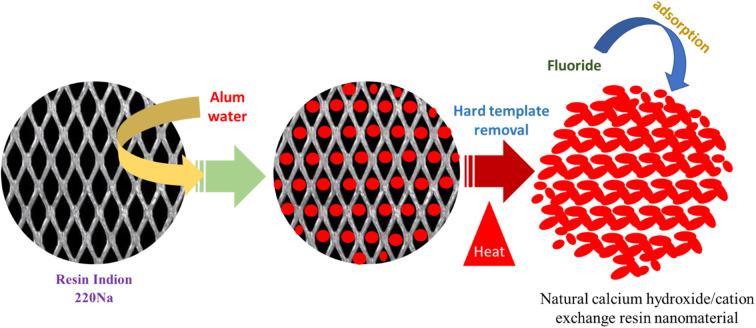

## 1. Introduction

Water pollution is becoming increasingly serious, and according to WHO, about 80% of diseases occur due to poor-quality drinking water around the world.^1^ Fluoride contamination in drinking water accounts for 65% of all the causes of dental fluorosis,^2^ although it is also an essential trace element for living organisms. In Vietnam, the acceptable fluoride content is 0.7–1.5 mg L^−1^, as regulated by the National Technical Regulation on Domestic Water Quality (QCVN 01-1:2018/BYT). Excessive or insufficient fluoride exposure can have negative impacts on the human health, such as bone and tooth diseases, decreased thyroid function, or brain damage. According to research, many areas in Vietnam contain high fluoride contents in water;^1^ thus this fluoride issue must be mitigated for public health protection.

Currently, fluoride can be treated using a variety of techniques. For example, flocculation–precipitation with alum and lime is widely used but generates a large amount of secondary sludge that needs further treatment. Other methods such as membrane filtration, reverse osmosis, electrochemistry, and ion exchange are also popularly used, which offer removal efficiencies of over 90%, but their operating costs are high and are affected by many other factors.^3,4^ Among various methods, adsorption is a cost-effective option that produces high-quality water after treatment, is simple to use, and is readily available with a variety of commercial forms of adsorbents.^5,6^ Several types of adsorbents have been reported to have high fluoride removal efficiency, including activated aluminum, activated carbon, activated iron, titanium composite, polymer resins, and organic adsorbents of biological origin.^6^ Recent studies have reported that the use of different metal oxide and hydroxide materials, such as iron, aluminum, and calcium, especially at the nanoscale, results in relatively high fluoride removal efficiencies in water owing to their high surface area, wide pH range of activity, and the ability to remove fluoride at low concentrations.^7–12^

In the Mekong Delta (Vietnam), the problem of naturally contaminated water from acid sulfate soil (popularly known as “alum water”) significantly impacts the quality of domestic water sources and resident health in this area. This alum water contains Fe, Al, Ca, Mg, and Si, which could be valuable sources of natural elements for various purposes. Recently, Fe and Al elements in this alum water were used for synthesizing iron oxide nanoparticles, which were then used as a catalyst in the heterogeneous Fenton reaction in textile dyeing wastewater treatment.^13^ The separation and application of other metals in this alum water have not been reported.

In this study, a pre-designed cation exchange resin (CER) column was first tested to remove calcium in naturally contaminated water from acid sulfate soil for water supply purposes and further used as a novel and natural calcium hydroxide-supported CER composite (NCHO/CER) for the treatment of fluoride in water. The results from EDX, XRD, FTIR, and SEM-mapping analyses were used to characterize the material. The kinetics, isotherms, and thermodynamics were also studied to understand the fluoride adsorption process. The effects of various operational factors on fluoride adsorption were investigated to determine the optimum adsorption condition, which was then verified by the treatment of fluoride in tap water, groundwater, and wastewater. This study could provide a promising environmental solution in utilizing the waste from spent resins after treating alum water as adsorbents for fluoride, and possibly for other pollutants in the aquatic environment.

## 2. Materials and methods

### 2.1. Chemicals and material synthesis

Chemicals such as KBr, H_2_SO_4_, HNO_3_, HCl, NaOH, KCl, fluoride standard solution (1000 mg L^−1^), SPADNS (C_16_H_9_N_2_Na_3_O_11_S_3_), and ZnOCl_2_·8H_2_O were purchased from Merck (Germany). Other chemicals were of analytical grade. Two types of resins in the ion exchange system were 225H and 220Na resins from Indion (India).

Natural calcium hydroxide/cation exchange resin nanomaterial (denoted as NCHO/CER) was synthesized from a spent cation resin column after ion exchange with natural alum water containing high calcium contents.^14,15^ The process of synthesizing the materials is as follows. In the first step, alum water was collected directly from canals in the Tri Ton district, An Giang province, Vietnam, and stored according to the standard of TCVN 6663-1:2011, and the pretreatment of water samples before analysis was carried out according to TCVN 6663-3:2016 (ISO 5667-3:2012). In the next step, Indion 220Na and Indion 225H resin beads were washed with distilled water and allowed to dry naturally and then placed in the column. The ion exchange column is designed with a column height of 15 cm, column diameter of 1 cm, resin weight of 5 g, and water flow rate of 3 L h^−1^. The alum water then flowed through the column containing Indion 220Na plastic beads to remove the iron and aluminum components from the water. The pretreated water flowed through the column containing Indion 225H resin beads with a flow rate of 3 L h^−1^ for 2 h to capture Ca^2+^ ions in the tiny pores of the resin. The cation concentrations in the initial and post-filtering alum water samples from each resin column were analyzed to evaluate the filtration efficiency (Table 1). The spent resin beads were then separated from the solution and stirred at a speed of 150 rpm with 2 M NaOH solution at room temperature for 15 min to precipitate CaO–Ca(OH)_2_ in the pores of the resin. After filtration, the solids were washed with distilled water and dried to obtain the NCHO/CER material.

**Table 1:** Efficiency of cation filtration *via* the pre-treatment process

Sample	Fe (mg L^−1^)	Al (mg L^−1^)	Cu (mg L^−1^)	Ca (mg L^−1^)	Mg (mg L^−1^)
Raw natural alum water	63.1	11.98	Non-detect	290.5	201.0
Effluent from the Indion 220Na resin bead column	Non-detect	1.22	Non-detect	255.8	183.5
Effluent from the Indion 225H resin bead column	Non-detect	Non-detect	Non-detect	Non-detect	96.7

### 2.2. Fluoride adsorption experiment

In a typical test, 50 mg of the NCHO/CER material was added into a 100 mL beaker containing 50 mL of 10 mg L^−1^ fluoride solution. The mixture was stirred well at room temperature (∼30 °C). After the survey period, the mixture was filtered through filter paper (*Φ* 11 μm) to collect the filtrate. The fluoride concentrations in water samples before and after adsorption were analyzed to evaluate the adsorption efficiency and capacity of the material. The factors influencing the fluoride removal in water consist of reaction time, initial solution pH, material mass, temperature of the reaction medium, and initial fluoride concentration. The experiments were arranged in a completely randomized design and repeated 3 times. The fluoride removal efficiency (*H*, %) and fluoride adsorption capacity (*q*, mg g^−1^) were calculated using eqn (1) and (2), respectively.1
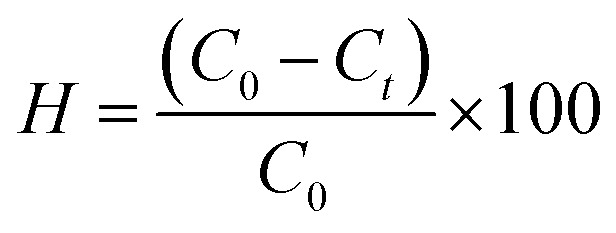
2
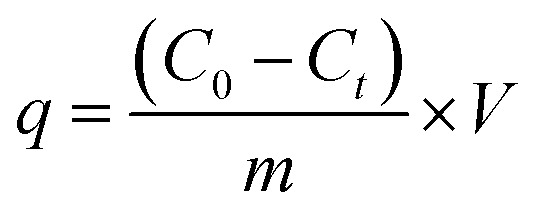
*C*_0_ and *C*_*t*_ are the initial concentration and concentration at time *t* of fluoride (mg L^−1^). *V* is the volume of sample (L) and *m* is the mass of the material (g).

The data from the adsorption kinetics study were then fitted with pseudo-first-order and pseudo-second-order kinetic equations.^16^ The Langmuir and Freundlich models were used to describe the fluoride adsorption isotherms.^17^ The thermodynamic parameters of the adsorption process were calculated according to the standard Gibbs free energy Δ*G*_0_ (kJ mol^−1^).^18^

### 2.3. Characterization and analysis

Calcium concentration in alum water samples was measured using the flame atomic absorption spectroscopy (AAS) method according to SMEWW (Standard Methods for the Examination of Water and Waste Water) – 3111B:2017. The hardness analysis method was according to TCVN 6224:1996 (ISO 6059:1984 (E)). Fluoride concentration was analyzed using a colorimetric method on a spectrophotometer according to SMEWW – 4500-F, D:2017. The material morphology and chemical composition were determined using scanning electron microscopy and energy-dispersive X-ray spectroscopy (SEM and EDX, JCM 7000, JEOL). The crystalline structure of the material was studied by X-ray diffraction (XRD, Aeris XRD, Malvern Panalytical). The surface functional groups were examined by Fourier transform infrared spectroscopy (FTIR, Alpha, Bruker).

## 3. Results and discussion

### 3.1. Material properties

**Fig. 1:**
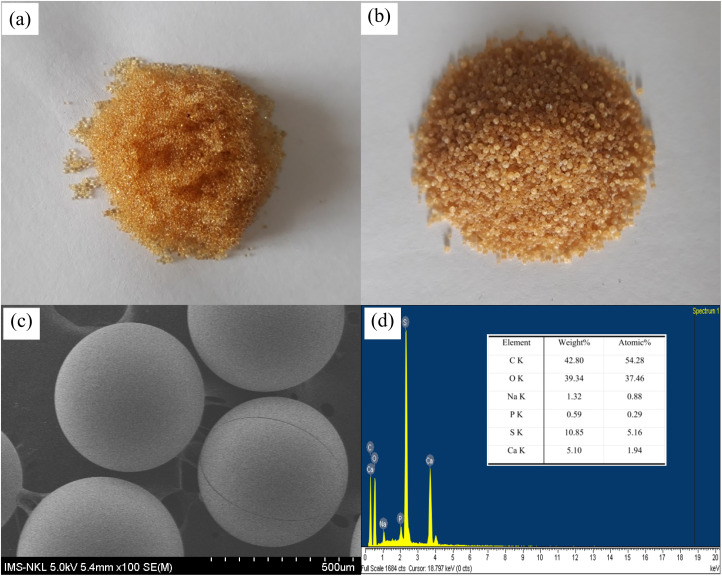
Images of (a) CER and (b) NCHO/CER materials; (c) SEM images and (d) EDX spectra of NCHO/CER materials.

Fig. 1 describes the morphology and chemical composition of the materials. The CER and NCHO/CER material in Fig. 1(a and b) display a remarkable difference in color, texture, and shape. Specifically, the color of the sample changed from the yellow of the original CER to a more silvery-white color in NCHO/CER, and the resin particle size of NCHO/CER was also larger than that of CER. The difference in color and shape indicates the existence of other forms of compounds in the pores of the CER resin. The SEM results of the material show that the basic physical structure of the NCHO/CER material did not change as compared to the original structure of the CER resin (Fig. 1(c)). In addition, the EDX results in Fig. 1(d) indicate that the NCHO/CER material consisted of 6 elements with predominant C (42.80%), followed by O (39.34%), and others such as Ca (5.10%), Na (1.32%), P (0.59%), and S (10.85%). The presence of some other elements is possibly due to their adsorption after the treatment of alum water.

**Fig. 2:**
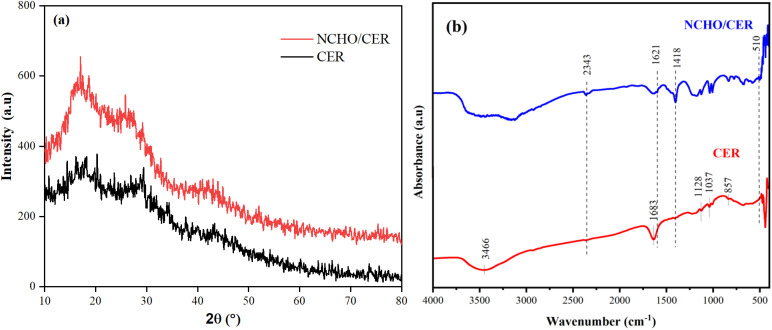
Results of (a) XRD and (b) FTIR analysis of CER and NCHO/CER materials.

As shown in Fig. 2(a), the diffraction peak at 2*θ* = 17.01° corresponds to the crystalline region. A subtle difference is observed in the XRD patterns of CER and NCHO/CER. Specifically, the intensity of the peak at 2*θ* = 17.01° is more pronounced in NCHO/CER than in CER. This increase in intensity may be attributed to the enlargement of the adsorbent particles, suggesting the presence of calcium forms on the resin surface.^19,20^ Before the FTIR analysis, the materials were dried at 100 °C and cooled to ambient temperature in a desiccator. Fig. 2(b) shows that the peaks of CER and NCHO/CER materials were significantly different. The peaks of CER at a wavenumber of 1037 and 1128 cm^−1^ were related to the sulfonic group (–SO_3_H),^21,22^ and the peak at a wavenumber of 1683 cm^−1^ was typical for the C=C double bond of styrene.^23^ The substitution of the benzene ring by the linkage of the sulfonic and divinylbenzene groups was determined by the vibration at a wavenumber of 857 cm^−1^, wavenumber range of 3577–3331 cm^−1^, and the intense peak at a wavenumber of 3466 cm^−1^ was the –OH bond.^21^ In the NCHO/CER material, the vibration at the wavenumber 510 cm^−1^ was typical for the Ca–O bonds,^24^ 1418 cm^−1^ for C–O bonding, 1621 cm^−1^ for the elongated vibration of water physically adsorbed on the CaO surface, and at 2343 cm^−1^ for adsorbed CO_2_ in the air.^25^

Based on the characterization results of the synthesized material, the synthesis mechanism of the NCHO/CER material can be described as follows. Cations, including Ca^2+^ and other ions (collectively referred to as M^*n*+^), in natural alum water are attached to the CER surface *via* an ion exchange reaction (eqn (3)). Subsequently, the NCHO particles will be formed in the pores of the CER resin particles when the resin, having undergone cation exchange with metal-contaminated water (R–SO_3_M^(*n*−1)+^), comes into contact with NaOH solution through the hydroxylation of metal ions (eqn (4)).

3 R–SO_3_H + M^*n*+^ → R–SO_3_M^(*n*−1)+^ + H^+^

4 R–SO_3_M^(*n*−1)+^ + NaOH → R–SO_3_Na + M(OH)_*n*_

### 3.2. Fluoride adsorption experiments

**Fig. 3:**
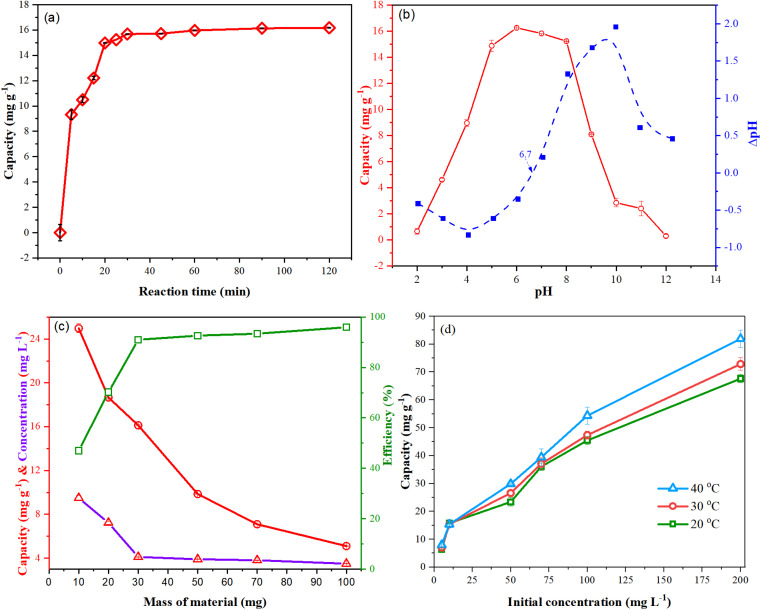
Effect of (a) reaction time, (b) solution pH, (c) mass of the material, (d) temperature and fluoride concentration on fluoride adsorption capacity using NCHO/CER.

**Fig. 4:**
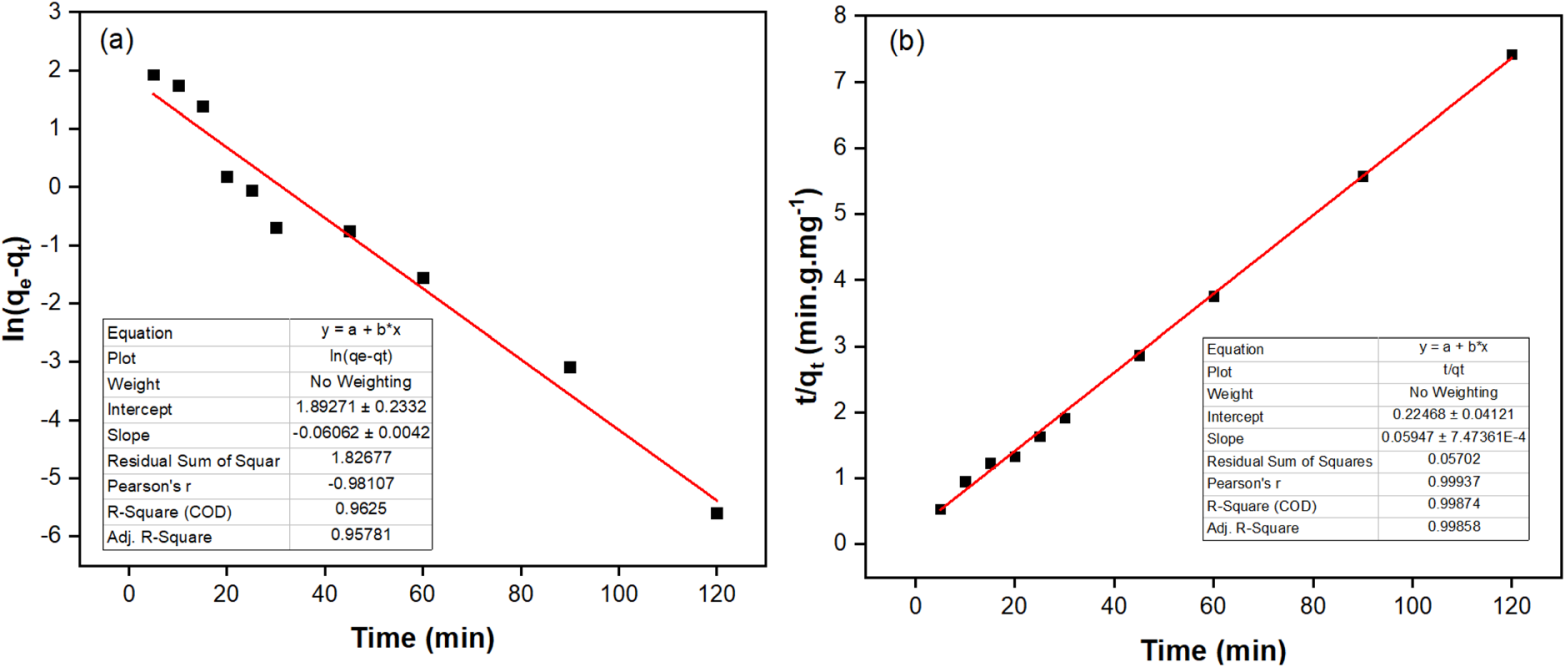
Fits of the experimental to kinetic models: (a) pseudo-first-order and (b) pseudo-second-order of fluoride adsorption on the NCHO/CER material.

Fig. 3(a) presents the adsorption of fluoride by NCHO/CER for 120 min. The fluoride adsorption capacity increased rapidly in the first 20 min, then slowly increased and reached an equilibrium state after 30 min. This trend can be explained by the fact that at the beginning of the adsorption process, the number of fluoride ions adsorbed on the surface of the material was still small; hence, the interaction force between the adsorption centers and the surrounding fluoride ions was very strong. When the amount of fluoride adsorbed on the surface of the material became dense, the force was adequately reduced until this material could not adsorb more, and then reached saturation after 30 min of the experiment. The fluoride adsorption kinetic models were built based on two models, including pseudo-first- and pseudo-second-order kinetic equations, as summarized in Table 2 and Fig. 4. Among the kinetic models, the fluoride adsorption kinetic data fitted well with the pseudo-second-order one because of its higher correlation coefficient (*R*^2^ = 0.9981), which is similar to the results in the literature.^16,17^ Accordingly, the theoretical fluoride adsorption capacity (*q*_e-TH_) was 16.64 mgF^−^ g^−1^, which is close to the experimental fluoride adsorption capacity (*q*_e-EX_ = 15.68 mgF^−^ g^−1^). Based on the adsorption equation, the apparent rate constant of pseudo-second-order kinetics was 0.0199 g mg^−1^ min^−1^. These findings prove that the adsorption process is primarily controlled by chemisorption rather than the physisorption mechanism.^26^

**Table 2:** Parameters of the pseudo-first- and pseudo-second-order kinetic equations of the fluoride adsorption process using the NCHO/CER material

Fluoride initial concentration (mg L^−1^)	*q*_e-EP_ (mg g^−1^)	Pseudo-first-order			Pseudo-second-order			Ref.
		*k*_1_ (min^−1^)	*q*_e-TH_ (mg g^−1^)	*R* ^2^	*k*_2_ (mg g^−1^ min^−0.5^)	*q*_e-TH_	*R* ^2^	
10	15.68	0.00194	64.10	0.9625	0.01990	16.64	0.9981	This study
10.2	15.48	0.2426	15.22	0.828	0.0638	15.46	0.9949	16
12	5.70	0.00241	9.036	0.826	−5.291	5.677	0.9961	17

pH is an essential factor that determines the adsorption capacity of materials because it affects the surface charge of the material and the ionization and characteristics of the pollutants.^18^ As shown in Fig. 3(b), the NCHO/CER material achieved an adsorption capacity greater than 14 mg g^−1^ at pH 5–8, with the highest capacity of approximately 16 mg g^−1^ at pH 6–7. At pH < 5, the fluoride adsorption efficiency was low due to the high H^+^ concentration hindering the fluoride adsorption process. Besides, there may be a competition for adsorption between the fluoride and SO_4_^2−^ added from the pH adjustment process using the H_2_SO_4_ solution. At pH > 8, the fluoride adsorption capacity also decreased, as at these pH values, the concentration of the OH^−^ anion gradually increased, leading to a competitive adsorption between the fluoride and OH^−^ anions for the adsorption centers of the material. These results are consistent with those reported in the literature.^18,27^ In addition, the isoelectric point (pH_pzc_) of the NCHO/CER material was determined to be 6.7. Accordingly, the material surface was positively charged at pH < 6.7; thus, the fluoride (F^−^ anions) adsorption process is based on the electrostatic interaction mechanism. However, at pH > 6.7, the adsorption capacity decreased significantly due to the negatively charged material surface and the competitive adsorption between the fluoride and OH^−^ ions.^28^ Thus, a slightly acidic to neutral environment (*e.g.*, pH 6–7) is suitable for fluoride adsorption by the NCHO/CER material.

As seen in Fig. 3(c), the fluoride adsorption capacity of the NCHO/CER materials is inversely proportional to the mass of the material, and it decreased with increasing material mass. On the contrary, on increasing the material mass, the treatment efficiency increased, resulting in a lower fluoride concentration after adsorption. This trend is reasonable since an increase in the adsorbent density result in a larger contact area and active sites available for fluoride adsorption. However, with a fixed amount of fluoride ions in the solution, the adsorption capacity decreased when the adsorbent mass increased. The determination of material mass depends on the fluoride concentration of the solution and the required fluoride output concentration. For a 10 mg L^−1^ fluoride solution, the output concentration reached 1.0 mg L^−1^ when using 30 mg of the material, meeting the water requirements for domestic use (QCVN 01-1:2018/BYT). In terms of adsorption capacity, when the material mass was lower than 30 mg, the capacity decreased rapidly, but the reduction was not significant for material weights greater than 30 mg. Therefore, a material mass of 30 mg was chosen for the subsequent investigation.

As the initial concentration and ambient temperature may have a significant influence on the adsorption capacity of materials, the adsorption capacity and efficiency under different solution temperatures and fluoride concentrations were investigated, as depicted in Fig. 3(d). Accordingly, the adsorption capacity tended to increase with the increase in the temperature and initial fluoride concentrations. The effect of initial fluoride concentration would be explained by the increase in these concentrations, possibly leading to an increase in the interaction between the fluoride ion and the adsorption centers on the material surface. At the same time, as the temperature increases, the fluoride ions in the solution would be more mobile, thus increasing the frequency of collisions between the material surface and the fluoride ions. This can be clearly observed when the solution temperature increased from 20 to 40 °C with a fluoride concentration of 70 mg L^−1^.

**Fig. 5:**
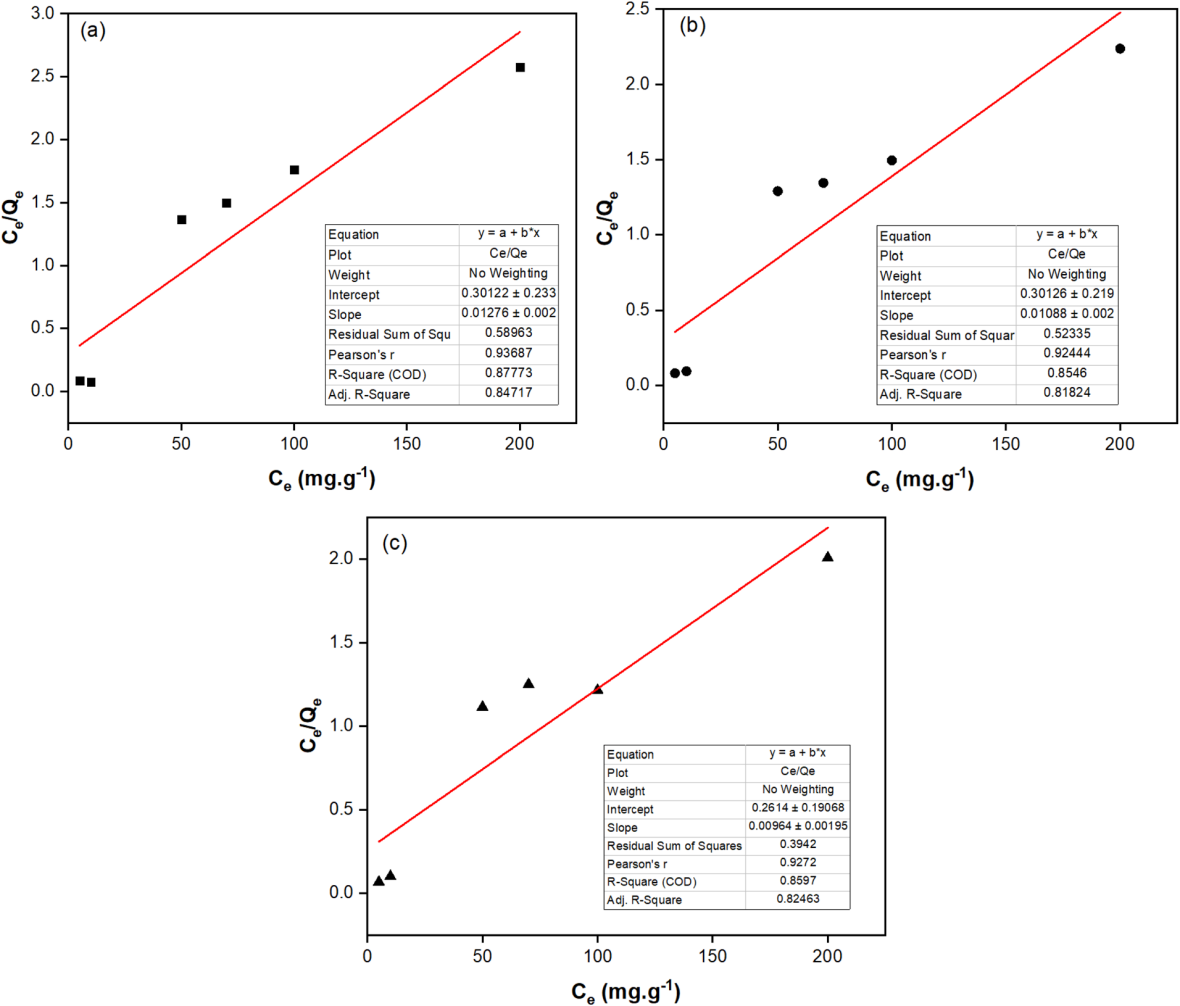
Langmuir isotherm model at (a) 20 °C, (b) 30 °C, and (c) 40 °C of fluoride adsorption on the NCHO/CER material.

**Fig. 6:**
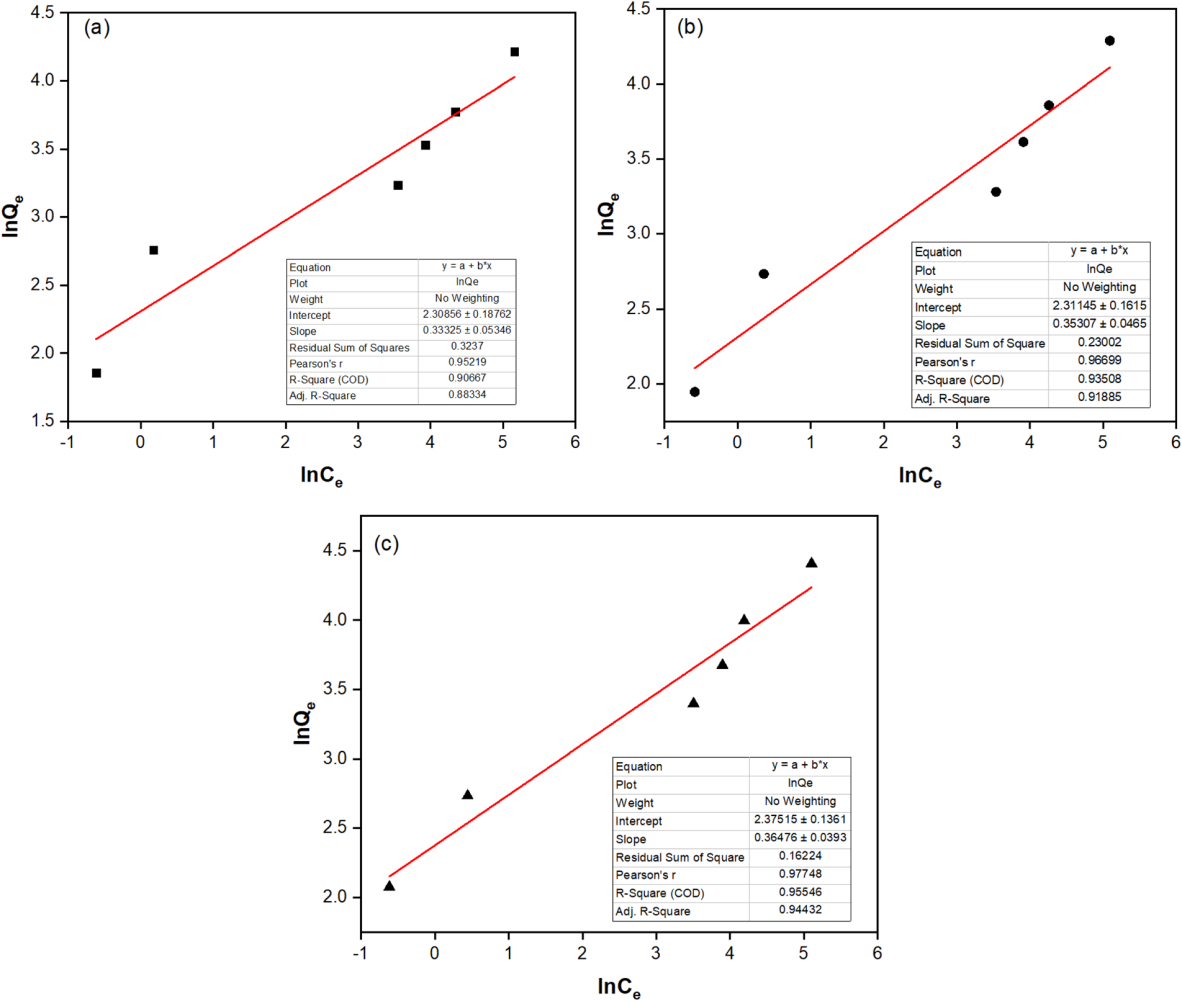
Freundlich isotherm model at (a) 20 °C, (b) 30 °C, and (c) 40 °C of fluoride adsorption on the NCHO/CER material.

The Langmuir and Freundlich isotherm models were built to describe the nature of the adsorption process, as given in Table 3, Fig. 5 and 6. Although both models can be used to describe the fluoride adsorption of the NCHO/CER material, the Freundlich model proved more suitable because of its higher correlation coefficient *R*^2^ (*e.g.*, 0.9351 at 30 °C) compared to that of the Langmuir isotherm model (*e.g.*, 0.8546 at 30 °C). The correlation coefficients of the two models are relatively high, especially the former, showing a favorable uneven adsorption process on a heterogeneous surface. The value of *n* from the Freundlich isotherm equation was in the range of 2.7–3.0, indicating that the NCHO/CER material is favorable for the adsorption of fluoride with the primary mechanism being a chemical sorption process.^29,30^ The primary fluoride removal mechanism may involve CaO anchored on the CER surface, which reacts with fluoride ions to form a more stable CaF_2_ after fluoride adsorption.^31,32^

**Table 3:** Parameters for Langmuir and Freundlich isotherm models

Model	Parameter	Temperature		
		20 °C	30 °C	40 °C
Langmuir	*Q*_max_ (mg g^−1^)	71.429	78.125	89.286
	*K*_L_ (L mg^−1^)	0.0319	0.0315	0.0304
	*R* ^2^	0.8777	0.8546	0.8597
Freundlich	*K*_f_ ((mg g^−1^) (L mg^−1^)^*n*^)	0.0994	0.0991	0.093
	*n*	3.000	2.832	2.741
	*R* ^2^	0.9067	0.9351	0.9555

We further compared the *Q*_max_ adsorption capacity obtained from this study with those from related reports.^29,30,33–43^ As shown in Table 4, although under different material synthesis conditions, the NCHO/CER material in this study provided higher adsorption capacities. Moreover, the NCHO/CER material was generated from a waste product of other water treatment processes and can be considered an environmentally friendly and low-cost material, which has great potential for practical water treatment applications.

**Table 4:** Comparison of fluoride adsorption capacities of some reported materials

Adsorbent	Temperature (K)	Contact time (h)	*Q*_max_ (mgF^−^ g^−1^)	Ref.
Phosphoric-modified limestone powder	298 ± 1	3	6.45	29
CaCO_3_	303 ± 1	1	12.5	30
Activated alumina			1.2	
Sugarcane ash			10.99	
Resin γ-alumina nanomaterial	303 ± 2	1.17	32.0	34
Diatomite	Room temperature	3	1.67	35
Sulfonic acid functionalized graphene oxide	313	1	4.26	36
γ-Fe_2_O_3_ nanocomposite	300	1	55.56	37
Fe_3_O nanoparticle-coated polyurethane foams	303	1.33	34.48	38
Al_2_O_3_ nanoparticle-coated polyurethane foams			43.47	
Zirconium loaded on spent resin powder	303	24	37.62	39
Zirconium-impregnated anion exchange resin	303	6	12.03	40
Ce/Ti oxide	Room temperature	1	44.37	41
Al-zeolite	303	2	1.999	42
Magnesium oxide-coated nanoparticles	301 ± 1	2.5	10.96	43
Indion 225H resin (CER)	303	2	0.04	This study
NCHO/CER	303	2	78.1	

Regarding the thermodynamics of adsorption, parameters such as Gibbs free energy (Δ*G*), entropy (Δ*S*), and enthalpy (Δ*H*) were calculated from the equilibrium constant at different temperatures (293, 303, and 313 K), as given in Table 5. In general, fluoride adsorption at 20 °C had a Δ*G* value of −14.57 kJ mol^−1^, which was less than zero, indicating that it occurred spontaneously under standard conditions. Furthermore, the Δ*G* value decreased to −15.04 kJ mol^−1^ when the temperature increased to 40 °C, suggesting that fluoride adsorption on NCHO/CER materials was more favorable at higher temperatures. In addition, the value of Δ*H* being −7.68 kJ mol^−1^ proved that the fluoride adsorption was an exothermic process (Δ*H* < 0) and can be considered as physical adsorption (Δ*H* < 40 kJ mol^−1^). The value of Δ*S* of 0.02 kJ mol^−1^ K^−1^, which was bigger than zero, indicated that the random solid/liquid interaction in the fluoride adsorption process was increasing. It also suggested an increased disturbance in the system due to altered hydration of fluoride ion adsorption.^30,44,45^

**Table 5:** Thermodynamic parameter for fluoride adsorption on NCH/CER

Δ*G* (kJ mol^−1^)	Value
293 K	−14.57
303 K	−14.80
318 K	−15.04
Δ*H* (kJ mol^−1^)	−7.68
Δ*S* (kJ mol^−1^ K^−1^)	0.02

**Fig. 7:**
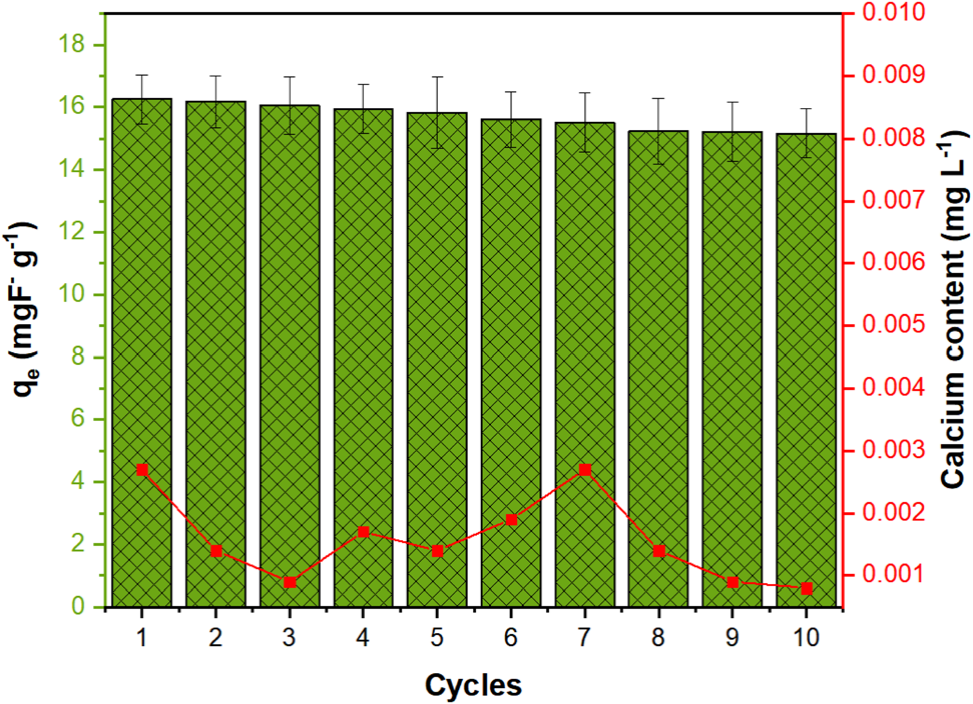
Regeneration study of the NCHO/CER material.

The recyclability of an adsorbent is a crucial factor in evaluating its industrial applicability, influencing both process efficiency and cost. After each cycle, NCHO/CER was separated using a centrifuge and reused for ten consecutive fluoride ion removal cycles (Fig. 7). The attained results described the fluoride adsorption capacity decreased from 16.26 to 15.17 mg g^−1^, retaining 93.3% of the initial capacity. These findings highlight the stability of NCHO/CER in the adsorption of fluoride over multiple cycles, indicating its potential for practical applications. The regeneration process was achieved by treating NCHO/CER with a 1 N NaOH solution. Besides, the calcium content was analyzed to determine the leaching of precipitate ions in the effluent or filtered media due to fluoride adsorption. These findings indicated an insignificant amount of calcium leakage from the material into the water.

To prove the practical applicability of the NCHO/CER adsorbent, fluoride adsorption tests were conducted for tap water and groundwater (data not shown). The experiments were carried out under unadjusted pH conditions, with a material mass of 1 g L^−1^ for 30 min at room temperature (30 °C). The tap water samples were randomly taken from the area of Long Xuyen city (An Giang province). The fluoride concentration in the tap water fluctuated around 0.857 ± 0.04 mg L^−1^. After the adsorption process, the remaining fluoride concentration was 0.034 ± 0.01 mg L^−1^, with the adsorption capacity of 1.29 mg g^−1^. Groundwater samples were taken from domestic wells in Ninh Xuan commune (Ninh Hoa town, Khanh Hoa province), where the fluoride concentration was considered to be at high levels. Fluoride concentration in the water samples was 5.13 ± 0.03 mg L^−1^, exceeding the standard QCVN 08-MT:2015/BTNMT. After adsorption, the fluoride concentration in the water was only 1.18 ± 0.15 mg L^−1^, corresponding to the adsorption capacity of 6.37 ± 0.09 mg g^−1^. After treatment, the fluoride concentration in groundwater reached the allowable limit for water supply (QCVN 01-1:2018/BYT). This verification hence proved the ability of NCHO/CER as a fluoride adsorbent in real applications.

## 4. Conclusions

Calcium from the water of acid sulfate soil was proactively recovered and successfully used as an effective adsorbent for fluoride removal in water. The waste-derived material showed an excellent fluoride adsorption performance with an adsorption capacity of 15.68, 1.29, and 6.37 mg g^−1^ in deionized water, tap water, and groundwater, respectively. Moreover, the operating conditions of the system, including room temperature and pH from 6 to 7, would be feasible for practical applications of this material. The adsorption process showed an exothermic (Δ*H* < 0) and physicochemical adsorption, which fitted well with the Freundlich isotherm model and the pseudo-second-order kinetics. The fluoride adsorption on NCHO/CER (with Δ*G* < 0) occurred spontaneously under standard conditions and was more favorable at higher temperatures. The NCHO/CER material was stable for ten consecutive removal cycles, retaining 93.3% of the initial capacity. Since waste–treat–waste is one of the important techniques that contribute to the circular economy, the solution provided in this study is a promising approach for practical water treatment applications to achieve sustainable development goals in the future.

## Data availability

The experimental data used to support the findings of this study are included in the manuscript. Other data are available from the corresponding author upon request.

## Conflicts of interest

The authors have no relevant financial or non-financial interests to disclose.
